# Bacterial rose garden for metagenomic SNP-based phylogeny visualization

**DOI:** 10.1186/s13040-015-0045-5

**Published:** 2015-03-21

**Authors:** Dmitry Alexeev, Tanya Bibikova, Boris Kovarsky, Damir Melnikov, Alexander Tyakht, Vadim Govorun

**Affiliations:** 1 Research Institute of Physico-Chemical Medicine, Malaya Pirogovskaya 1a, Moscow, 119435 Russia; 2 Moscow Institute of Physics and Technology, Institutskii Per. 9, Moscow Region Dolgoprudny, 141700 Russia; 3 Data Laboratory, Moscow, Russia

**Keywords:** Metagenomic data visualization, Rose garden, Gut microbiota, Phylogeny visualization

## Abstract

**Background:**

One of the most challenging tasks in genomic analysis nowadays is metagenomics. Biomedical applications of metagenomics give rise to datasets containing hundreds and thousands of samples from various body sites for hundreds of patients. Inherently metagenome is by far more complex than a single genome as it varies in time by the amount of bacteria comprising it. Other levels of data complexity include geography of the samples and phylogenetic distance between the genomes of the same operational taxonomic unit (OTU). We have developed the visualization concept for the representation of multilayer metagenomics data – the bacterial rose garden. The approach allows to display the taxonomic distance between the representatives of the same OTU in different samples and use variety of the metadata for display.

**Results:**

We have developed the principle of visualization allowing for multilayer information representation. We have incorporated data on OTU diversity across metagenomes and origin of the samples. The visual representation we have called “rose” is focused on the phylogenetic distance between the representatives of the same OTU. The visual representation is realized as interactive data chart which allows user to interact with data and explore variables. It is known that classical representation of the taxonomic tree is a reduction of information from original pairwise distance matrix. The visualization presented is a way to save all the information available through projection of distance matrix into single dimensional space of one sample. It could serve as a basis for further more complex information representation. We have used the principle proposed for visualization of 101 bacterial OTUs phylogenetic distances, finally we provide open code for the web page generation.

**Conclusions:**

Bacterial rose garden is a versatile visualization principle coping with the major difficulties of metagenomic big-data visualization without loss of data. The method proposed is showing the interconnectedness of variables and is realized as user-friendly web page allowing for dynamic data exploration. The concept provided serves as one of the original approaches for metagenomic data representation and sharing. Full functional prototype could be found at http://rosegarden.datalaboratory.ru

**Electronic supplementary material:**

The online version of this article (doi:10.1186/s13040-015-0045-5) contains supplementary material, which is available to authorized users.

## Background

One of the recent sources of genomic information is metagenome. The techniques for metagenomic sequencing are becoming more and more robust and as a result, we produce a much higher volume of information in smaller periods of time.

One of the most studied metagenomes is that of human gut. It is considered as one of the most complex - containing tens of millions genes [1] and over several hundred bacterial species [2]. It concedes only to complexity of soil metagenome [3]. Additional value of human gut microbiome studies is its relation to the health and disease issue [4-6]. High interest of a scientific society in theranostic potential of gut microbiome [7] would surely lead to generation of more and more datasets in addition to thousands generated until today.

Metagenomic data processing has nuances specific for such datasets. Variety of scientific groups and several major consortia produce and support regular renewal of public metagenome analysis tools [8-10], most of those performing two main steps - metagenomic reads processing and further clustering or classification. Read processing is mostly done through mapping, when reads are mapped to catalogues of genes or genomes. If catalog being used covers most taxonomic groups presented in metagneom then the mapping is for the most cases sufficient for analysis as all gut metagenomes are alike and differ only in a small part of genomic sequences (mainly single nucleotide polymorphisms, SNPs) [11]. The data being produced by mapping are multidimensional matrices, representing the number of each OTU or gene per sample. Each sample is a vector of size up to several hundred for OTUs representation.

Metadata for the samples in human gut microbiome is vast and depends on data available from the clinic. Content can cover such aspects as geography of samples, age, sex and disease. It can also include sophisticated data on the results of biochemical analyses. The aim of the metagenomic data processing services is to define the interconnection between the properties of the metagenome and variables in metadata. This would lead to hypothesis generation on bacterial disease drivers or functional role of genes in metagenome.

It should be noted, however, that multidimensional data of this kind is very sensitive to preacquisition steps. Anything can influence the result of metagenomic analysis: ranging from patients diet to DNA extraction methods and data processing. It is crucial to have versatile tools for visual data analysis which would provide the means for artifact and confounding factors discovery. In this aspect any additional novel visualization tool is of a great value to the community.

Most of the visualizations produced for metagenome analysis so far are based mainly on heatmaps or dimension reduction to 2D or 3D space, such as principal components analysis (PCA) or multidimensional scaling (MDS).

At the moment, a number of tools exist for visualization of metagenomic data: SynTView [12], MetaSee [13], MetaPhlAn [14] and others, most of them are concentrated on community profiling and use the phylogenetic information for visualizing relationship between OTUs. Some of them like SynTView allow for intra-OTU data analysis based on SNPs, however, none is using the full information on intra-OTU SNP data as a basis for data representation.

We have used the data from the three of the most large-scale metagenomic studies to calculate the phylogenetic distance between the genomes belonging to the same OTU represented in various metagenomes (of different subjects). After the distance was calculated we have worked on the data representation using the following data variables: the bacterial OTU (n = 101), sample (n = 196), pairwise distance (between each of the representatives of the OTUs in each of the 196 samples) and the geographic region of sample origin.

We have developed the web interface using D3.js allowing for interactive data exploration and filled it with the data described above. The interface presented is capable of any other data representation in the proposed format and, moreover, open code will allow the researchers to use it as a basis for more sophisticated visualization providing additional visual channels. In our paper we discuss the limitations of other methods for visualization of SNP data and provide use cases for the approach proposed.

## Methods

### Datasets

We have used the data for 436 samples of metagenomic DNA whole-genome sequencing (WGS) acquired in four countrywide studies (Table 1). The reference catalogue was composed of 353 genomes as described previously [15] with extra genomes added (Additional file 1: Table S1).

**Table 1:** **Data used in the study**

Country	Source	Number of samples	Number of donors and	Sequencing platform	Reads metrics
USA	Human Microbiome Project [21]	138	50 (single samples), 41 (two samples), 2 (three samples)	Illumina	101 bp, paired-end
Denmark	MetaHIT project [2]	85	85	Illumina	44 bp (13 samples), 75 bp (72 samples)
China	BGI-Shenzhen [4]	126	50 (type II diabetes), 70 (healthy), 6 (unknown)	Illumina	75 bp
Russia	Metagenome.ru consortia [15]	162	116(single sample), 2 (two technical repeats),14 (two samples and one technical repeat)	SOLiD	50 bp
Russia	Metagenome.ru consortia [15]	5	5	Illumina	100 bp

Origin of WGS metagenome data used in prototype.

### Reads mapping and distance calculation

The reads were preprocessed and mapped to the reference genome catalogue as described previously [15] (mapping statistics are shown in Additional file 2: Table S2).

Pileup files were acquired from the resulting BAM files using samtools; the procedure was performed separately for every OTU in every sample. For all the OTUs in samples with percentage of genome covered by reads over 50%, the consensus sequence was defined in the following way: the positions with coverage less than 8-fold were filled with gaps and the positions with coverage greater or equal than 8-fold were filled with the nucleotide supported by the most reads. In case of two or more variants with equal support, the random nucleotide was chosen among variants. The distance between the realizations of an OTU in two samples was defined as an edit distance between the two corresponding consensus sequences normalized by the overlapping sequence length excluding the gaps. The distances were calculated only for the sufficiently covered OTUs – having the number of positions overlapping across all the pairs of samples equal to or higher than 50,000. Using this definition, we have calculated the distance matrices for 101 OTUs (the remaining OTUs were not substantially represented in majority of the samples) totally including data from 196 samples (the remaining samples did not have enough data for distance calculations for all of the OTUs).

### Software implementation

Visualization is performed using D3.js. All installation and setup instructions are available on https://github.com/naxxateux/bacteria. Full functional prototype could be found at http://rosegarden.datalaboratory.ru.

## Results

### Problems to avoid

We have initially worked on data representation for individual OTUs. Here we had to display values for 196 × 196 symmetric distance matrix, as each representative of OTU in the sample had a value for pairwise distance. The most widely used approaches to sample distance visualization for the data of such kind are heatmaps, various 2D projections and trees.

**Figure 1:**
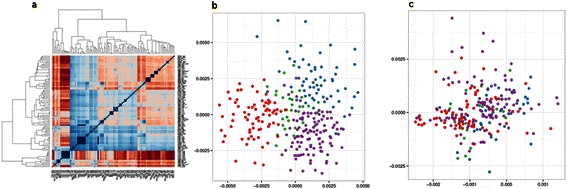
**Basic data visualization techniques for microbiota studies.** Examples of heatmap **(a)** and MDS **(b,c)** visualization of sample distances.

Heatmap (Figure 1a) is able to depict pair wise distances between samples using a color gradient. However, widely used heatmap has a major disadvantage: a linear measure (distance) is visualized via nonlinear color scale. Here colors should be interpreted all the time (e.g. blue means close, red means distant). Moreover, the interpretation of colors is not always unique [16]. Therefore, while the information is available, it could be hard to make conclusions, additionally it is redundant in the case of pair wise distances, as matrix is symmetric relative to diagonal.

MDS projection (Figure 1b, c) diminishes the dimensions of the problem under investigation from N × N to 2 (where N is the number of samples). Such methods (including PCA) are very popular for exploratory data analysis of the genomic data. Most frequently distances could be easily calculated using the metrics chosen and variety of packages provide functionality to build a 2D projection.

The method allows easy visual identification of clusters (Figure 1b) and further mine the data to discover the nature and the origin of the clustering.

The shortcoming of the 2D visualizations is the loss of information imminent with dimension reduction. The procedure of projection calculation does not guarantee the uniqueness of the representation and the results can be dramatically different (visually) for the very close distance matrices. This is especially problematic when the number of samples increases during the study. There is no sense in comparing old and new 2D projections. The core of the problem is absence of connection between the individual data point values and their coordinates on the axes.

Another representation of the distance data is a phylogenetic tree, however, the issue of multidimensional reduction from N × N space to a tree can potentially hide information crucial for the metagenomic studies. There also some artifacts (such as long branch attraction) in trees constructed using distance-matrix methods.

### Concept of a bacterial rose

**Figure 2:**
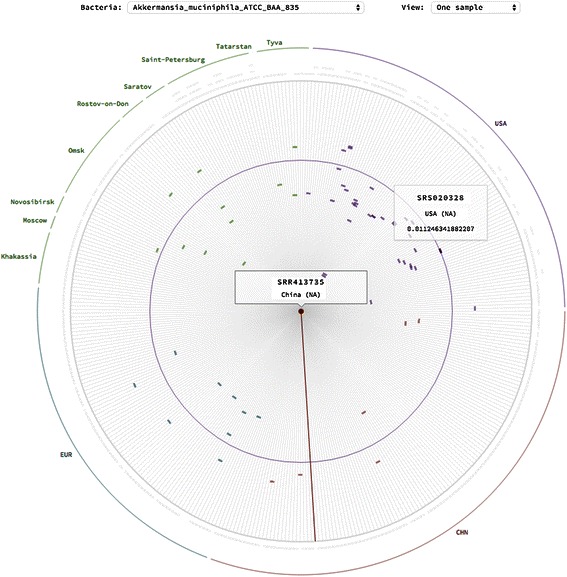
**Bacterial rose.** The bacterial rose visualization principle.

Most of the visualization tools rely on the habitual visualization principles. We had an idea of developing a new principle putting the above-discussed requirements in front. The essential decision was to use the distance between representatives of the same OTU in the samples as the major characteristic. Here we use a classical visual channel - position. For each separate bacterial genome (OTU), we base the visualization on the distances relative to the samples. We place a selected sample at the center of the circle and all the other samples are situated in a circle around it. The radial distances were chosen to represent the distance from the central sample to the samples on the circle. We have used the color channel to encode country of sample origin (USA, China, Europe or Russia). As the resulting picture reminded of a wind rose, we have named this visualization a bacterial rose (Figure 2).

We have further used the possibilities of interactive data visualization. The samples were grouped on the sectors of the circle according to a country of origin. As a user interacts with the visualization, mouse hover over any sample shows a hint with a sample name, region and exact distance value. Mouse click places the selected sample to the centre of the circle and all the other move according to the distance relative to new sample in the center.

Next feature would be to display the data for the whole ensemble of pair wise sample distances on a single picture. While one bacterial rose has N-1 values displayed on radii, the new picture has to display (N-1) × N values (where N is the number of samples).

**Figure 3:**
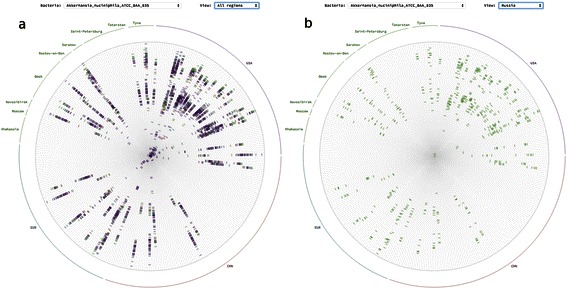
**Regional bacterial rose. a.** Bacterial rose of single OTU with all the representatives of this OTU from all the regions showing all the distances from the sample on the radius to all the other samples. **b.** The same for the chosen region, i.e. only distances from the samples on the radius to the sample belonging to chosen region are shown.

We have overlaid all the N roses so that every radius now displays the distance from the current sample on the radius to all the rest (Figure 3a).

The presented visualization allows to visualize the following properties of the data:

1. The occurrence of the selected OTU in the microbiota of the patients: radii are filled if OTU is present and empty otherwise.
2. The distribution of the distances from the sample on the radius to the rest of the samples: minimum maximum and distribution as a density on the radius
3. The regional dependence of the distance for the samples on the radius is visualized as color clustering along the radius
4. The pattern of the distribution for pair wise distances for the OTU is displayed by the complete image of the rose
5. Regional dependencies of the distance distribution could be additionally displayed on the regional rose (Figure 3b).

### Bacterial rose garden

**Figure 4:**
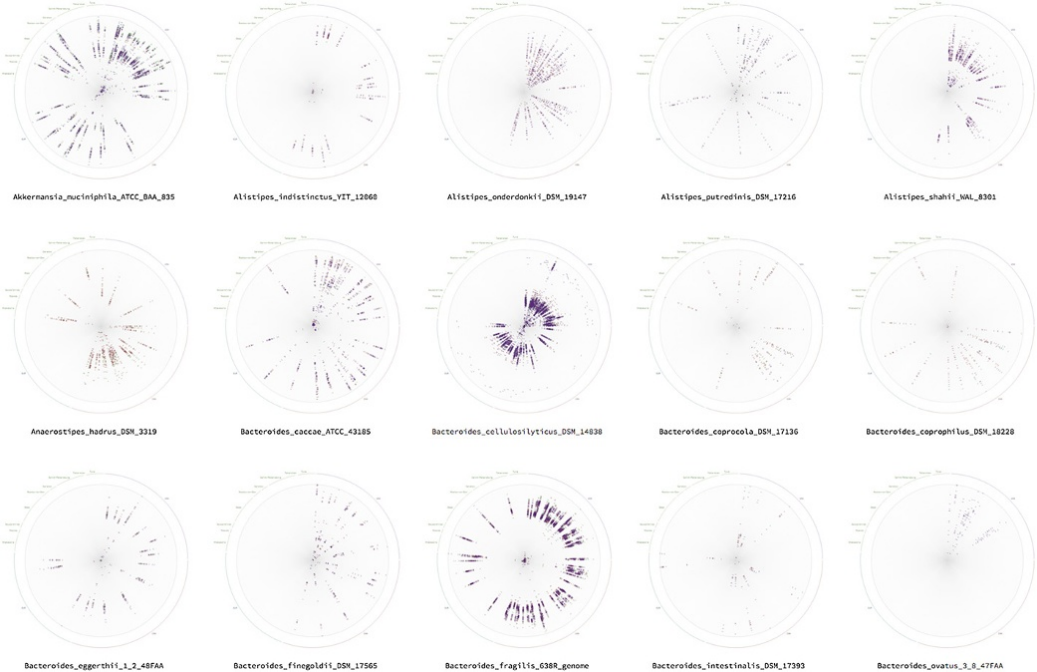
**Rose garden.** Part of the rose garden presenting all the bacterial roses for the chosen OTUs.

The rose described above could be created for every OTU in the study. To show the properties of the all the metagenomes in the study we have created miniatures of the separate roses on a single web page (Figure 4). The final picture reflecting the properties of all the OTUs in the study was called “Bacterial rose garden”. We have made every rose clickable allowing the researcher to turn to the single rose display for more thorough analysis. The rose described above could be created for every OTU in the study.

### Capacities of the visualization

Each visualization of the data normally allows to make some features of the dataset to be easily identifiable. Below we present several biological features of the dataset used as example, which were easily detected by presented visualization concept.

### Clustering by country

**Figure 5:**
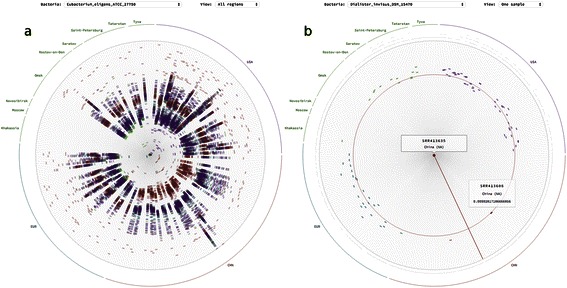
**Clustering and travelling rose. a.** The example of the *Eubacterium eligens* shows distinct clustering of samples from China and Russia and intermixed samples from Europe and USA. **b** - *Dialister invisius* as an example of visualization of the travelling bacteria, where in the circle of the closest Chinese sample radius we find lots of samples from Europe or USA, therefore concluding that distance calculated is smaller.

OTUs that show distinct clustering by region look as petals of a striped color on the “All regions” representation of the rose (or on the miniature picture in the rose garden). The order of stripes on the petal shows the between country distances (Figure 5a).

### Traveler bacteria

The quest for traveler bacteria was the initial motivation for data analysis – we hoped to find the bacteria in the metagenomes of one country which could have originated from another country according to phylogeny. Such an example is found in the Chinese samples; using the interactive visualization interface we can study the similarity closeness of the samples (Figure 5b). For one of the samples from China (in the center of the rose), the circle appearing on mouseover the closest sample from China contains several samples from Europe or US inside. The visualization could be interpreted as the fact that the bacterial species has come from Europe or USA recently. This conclusion is especially tempting, as the bacterial species *Dialister invisius* is often found in the mouth [17] and is not an obligate anaerobe – which gives it more chances to be transmitted. However, another interpretation could be the general lack of data on this species in the Chinese samples.

### Two distinct OTUs referring to a single reference genome

**Figure 6:**
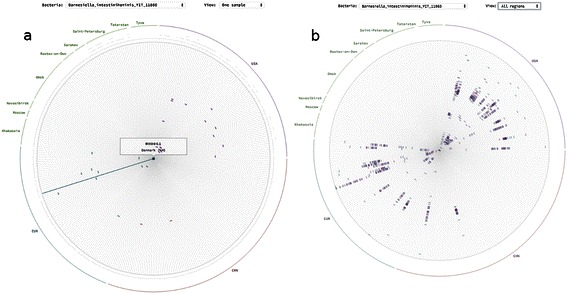
**Two OTUs case. a.** Single sample view of bacterial rose for *Barnesiella intestinihominis* showing several samples much closer than the others. **b.** All samples view showing distribution of the distances, two distinct groups could be identified – one with smaller phylogenetic distances and one with larger.

The species *Barnesiella intestinihominis* was recently discovered [18], and it is has not been well studied yet. According to the rose of the OTU, two distinct species could be distinguished. The samples are separated into two clusters where the intra-cluster distance is much smaller than distance between the clusters even though the regions are different (Figure 6).

### Quality control and artifacts

**Figure 7:**
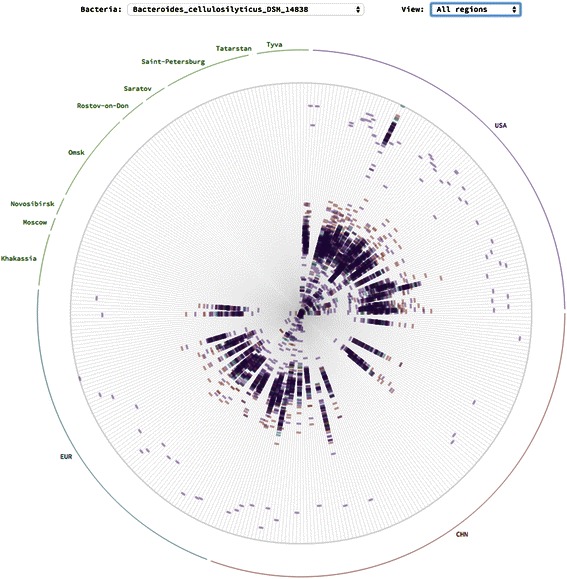
**Outlier example.** Unusual distance distribution for the sample SRS014979 of Bacteroides cellulosilyticus DSM_14838 OTU (top right corner outlier).

Sample SRS014979 for OTU *Bacteroides cellulosilyticus* DSM 14838 has unusual pattern for the distance distribution in the “All regions” view (Figure 7). This shows that its phylogenetic distance is much higher than the rest in the same OTU. It implies that this representative of OTU has around twice as many mutations in one of metagenomes from USA. This is a distinct signal to check the calculations or data quality manually.

### Dense garden

One of the beneficial representations is the top-level representation of the rose garden with a possibility to immediately go deeper into the data. In a dense rose garden, we can easily locate OTUs with higher or lower number of representatives and we can immediately see if the OTU has obvious region-specific clustering.

## Discussion

As it was brilliantly said by Nils Gehlenborg [19]: “The challenge is to create clear, meaningful and integrated visualizations that give biological insight, without being overwhelmed by the intrinsic complexity of the data”. The data from metagenomics field is clearly presenting a scientist with such a challenge. We are completely sure that developing a variety of visualization approaches and testing them on real data sets is the way of trial and error and the only way to extend our toolbox of visual data analysis.

We have planned this work with one objective in mind - search for new solutions in visualization knowing the specifics of the data in advance. All of the popular visualization techniques in the field of metagenomics were known and and used long before the metagenomic data appeared. Heatmaps and MDS approaches are standard ways to interpret multidimensional data. Here we let the multidimensionality along and try to model visulization according to the data features inportant in the study. Moreover, we use the concept of data-driven documents [20] proposed by M.Bostock et al. where the presented data is interactive which allows us to reduce multidimensionality of data and in fact every single picture represents a line in the matrix of N × N, where N is the number of samples. The “garden” approach where the thumbnails of single visualizations are collected on one page allow for representaion of M stacked matrices, where M is the number of bacteria we want to explore in each sample.

It is sure enough that the concept of visulization is lacking habitual informativity of heatmaps and scalings to 2D or 3D space. We are now on the way trying to overcome the utility of standard approaches. However, we show several cases where the invented bacterial rose visualization could help the researcher to identify important trends in the data and even to gain the biological insight - which is afterall the goal of visualization.

Clear overview of the samples, quality control, data clustering and outliers identification were the capabilities of the visualization demonstrated on the dataset. We believe that further development of this and other visulizations is the key to creative and brave biological interpretation of the results obtained in omics experiments.

## Conclusions

We have developed a novel visualization approach using interactive data techniques. The approach was tested on the real multidimensional metagenomic data and showed several promising possibilities for data exploration. We believe such approaches present a growing support of self-descriptive data publications in scientific articles.

## Additional files

Additional file 1: Table S1.
**List of genomes used as references for read mapping.**
Additional file 2: Table S2.
**Mapping statistics of metagenomic samples used in the study.**

## Abbreviations

MDS: Multidimensional scaling
OTU: Operational taxonomic unit
PCA: Principal components analysis
SNP: Single nucleotide polymorphism
